# Mortality after Use of Paclitaxel-Coated Balloons Correlates with Total Cumulative Dosage of Paclitaxel in Real-World Analysis

**DOI:** 10.3390/jcm10163747

**Published:** 2021-08-23

**Authors:** Seon-Hee Heo, Shin-Young Woo, Seung-Hyuk Choi, Taek-Kyu Park, Young-Soo Do, Kwang-Bo Park, Dong-Ik Kim, Young-Wook Kim, Yang-Jin Park

**Affiliations:** 1 Department of Surgery, Yonsei University College of Medicine, Seoul 03722, Korea; sunnyms@yuhs.ac; 2 Vascular Surgery, Department of Surgery, Heart Vascular Stroke Institute, Samsung Medical Center, Sungkyunkwan University School of Medicine, Seoul 06351, Korea; evering.woo@samsung.com (S.-Y.W.); dongik.kim@samsung.com (D.-I.K.); 3 Intervention Cardiology, Department of Internal Medicine, Heart Vascular Stroke Institute, Samsung Medical Center, Sungkyunkwan University School of Medicine, Seoul 06351, Korea; sh1214.choi@samsung.com (S.-H.C.); taekkyu.park@samsung.com (T.-K.P.); 4 Department of Radiology and Center for Imaging Science, Samsung Medical Center, Sungkyunkwan University School of Medicine, Seoul 06351, Korea; ys.do@samsung.com (Y.-S.D.); kbjh.park@samsung.com (K.-B.P.); 5 Department of Surgery, Kangbuk Samsung Hospital, Sungkyunkwan University School of Medicine, Seoul 03181, Korea; young52.kim@samsung.com

**Keywords:** exposure, mortality, paclitaxel, risk factor

## Abstract

This study used independent, real-world, patient-level data to examine whether the dosage or frequency of paclitaxel exposure correlated with mortality during follow up. We conducted a retrospective analysis of patients treated with a drug-coated balloon (DCB) for an atherosclerotic femoropopliteal lesion from February 2013 to December 2018, excluding patients with non-atherosclerotic lesions or restenosis after DCB treatment in another hospital. We investigated the causes of death, comorbidities (including cancer status), and the initial and total cumulative dosages and frequency of paclitaxel use. To determine whether the dosage or frequency of paclitaxel exposure affected mortality during follow up, we analyzed the risk factors for all-cause death by conducting a time-dependent Cox regression analysis that considered demographics, comorbidities, lesion and procedural characteristics, and paclitaxel exposure data (dosage and frequency). Our analysis examined 225 patients (mean age 71 ± 9 years, range 38–93 years, male 81%). During a mean follow-up duration of 35 months (range 1–89 months), 56 patients (24.9%) died from cardiac disorders (16%, including acute myocardial infarction, heart failure, or sudden cardiac arrest), malignancy (14.3%), respiratory failure with pneumonia (12.5%), septic shock (12.5%), or another cause. Univariable and multivariable Cox regression analyses identified age (hazard ratio, HR, 1.057; 95% confidence interval, CI, 1019–1096; *p* = 0.0032), critical limb ischemia (CLI) (HR, 4135; 95% CI, 2171–7876; *p* < 0.0001), and the total dosage of paclitaxel (mg) (HR, 1.040; 95% CI, 1006–1074; *p* = 0.0210) as predictors of mortality during follow up. The subgroup analysis found that the total dosage of paclitaxel (mg) was also a predictor of mortality during follow up in the CLI group (HR, 1.046; 95% CI, 1007–1087, *p* = 0.0198). The estimated cut-off value of total cumulative paclitaxel dosage for predicting mortality was 12 mg as evaluated by minimum *p* value approach. This patient-level analysis identified the total cumulative dosage of paclitaxel as a predictor of mortality after the use of paclitaxel-coated balloons. Our results provide limited information about the potential dose–response relationship underlying paclitaxel-associated mortality concerns.

## 1. Introduction

Drug-coated balloons (DCBs) were developed to improve the patency of percutaneous transluminal balloon angioplasty, minimizing neointimal hyperplasia by delivering an anti-proliferative agent directly to the angioplasty site [1]. Paclitaxel DCBs are the most widely used DCBs, and superior results in treating femoropopliteal lesions have been reported, compared with non-paclitaxel-coated devices.

However, in a recent systematic review and meta-analysis of randomized controlled trials (RCTs), Katsanos and colleagues reported an increased mortality risk two and five years after the use of paclitaxel-coated devices [2]. Subsequently, the U.S. Food and Drug Administration (FDA) also reported that study subjects treated with paclitaxel-coated devices might face an increased five-year mortality risk compared with patients treated with uncoated devices [3].

Katsanos’s results and the FDA’s meta-analysis had limitations, such as small sample sizes, missing data, and the inclusion of patients treated with different paclitaxel-coated devices, and neither report identified a pathophysiologic mechanism for the deaths. Several observational studies and a recently published RCT [4] found no evidence that femoropopliteal artery revascularization with drug-coated devices increased long-term mortality rates compared with non–drug coated devices [4,5,6,7,8].

So far, it is unclear whether paclitaxel-coated devices are associated with long term mortality, and the controversy is likely to continue until more evidence becomes available. An important issue is whether the potential risks of paclitaxel DCBs have a dose–response relationship. Therefore, we looked for a correlation between the dosage and frequency of paclitaxel exposure and mortality during follow up using real world, independent, patient-level data.

## 2. Methods

This is a retrospective analysis of all patients who underwent endovascular treatment for femoropopliteal occlusive disease using DCB angioplasty with or without stenting or atherectomy between February 2013 and December 2018. The study period for the cause-of-death investigation was from February 2013 to July 2020. De novo lesions; restenosis lesions after plain balloon angioplasty, atherectomy, or stenting; and stenosis of a bypass graft were all included in this study. Patients with a non-atherosclerotic lesion or restenosis after DCB use in another hospital were excluded. This study was approved by the Institutional Review Board of Samsung Medical Center (No. 2020-01-082), and informed consent was not required.

We reviewed patient demographics (age, sex), comorbidities (diabetes, hypertension, smoking history, coronary artery disease, chronic kidney disease, hyperlipidemia, cerebrovascular disease, chronic obstructive pulmonary disease, cancer), treatment indication (claudication or critical limb ischemia, CLI), lesion characteristics (Trans Atlantic Inter-Society Consensus, TASC classification), previous intervention history, and treatment details. The cancer status of patients was investigated when the cancer was newly diagnosed after the initial endovascular treatment with DCB or when it was in an active state that required treatment or follow up. Previous intervention history (stenting, atherectomy, plain balloon angioplasty, or surgical bypass for the treatment of a femoropopliteal lesion) was considered. All causes and dates of death were identified using data from the National Health Insurance Service, a telephone survey, and a medical record review, including death certificates. Complete information was gathered for all deaths during the study period, except for 4 patients with no telephone connection and 1 emigrant patient.

### 2.1. Treatment Details and Paclitaxel Dosage

All procedures were performed by vascular surgeons, cardiologists, or vascular interventionists. Two types of DCB (IN.PACT Admiral, Medtronic, Dublin, Ireland, 82.1%; Lutonix, Bard, Tempe, Arizona, 17.9%) were used at the treating physician’s discretion. The balloon diameter and length were selected using angiographic measurements of the proximal and distal normal segments. Most patients had vessel preparation using standard percutaneous transluminal angioplasty (PTA), and some patients with a calcified lesion underwent debulking atherectomy. Nominal inflation of the DCBs was maintained for about 2 min, in accordance with the DCB instructions for use (IFU). All patients were given a loading dosage of 300 mg of clopidogrel and 325 mg of aspirin before the procedure and were maintained on dual antiplatelet treatments for a month and then changed to a single antiplatelet treatment for a minimum of 6 months unless contraindicated.

To determine the correlation between the dosage or frequency of paclitaxel exposure and mortality, we investigated the initial and total dosages of paclitaxel and the frequency of DCB use. The nominal paclitaxel dosage per balloon was defined according to the product length and diameter, as described in the IFU. To calculate the initial paclitaxel dosage, the nominal paclitaxel dosage per balloon for each DCB used in the initial procedure was summed. The frequency of DCB use was defined as the total number of times that a DCB was used to treat restenosis of the same lesion or of other, separate steno-occlusive lesions during the follow-up period. To calculate the total dosage of paclitaxel, all paclitaxel dosages per balloon used during the follow-up period were added together.

### 2.2. Statistical Analysis

Statistical analyses were executed using SAS version 9.4 software (SAS Institute, Cary, NC, USA) and R version 3.6.2 (R Foundation for Statistical Computing, Vienna, Austria). For the basic descriptive statistics, continuous variables are presented as means and standard deviations, and they were compared by the Mann–Whitney test; categorical variables are described as frequencies and proportions, and they were compared by Fisher’s exact test and the chi-square test. The predictors of all-cause mortality during follow up were analyzed using Cox regression models (univariable and multivariable) that considered demographic variables, comorbidities (including cancer status), treatment indications, previous intervention history, and variables related to paclitaxel exposure (initial and total paclitaxel dosages, frequency of DCB use). The frequency of DCB use and total dosage of paclitaxel were dealt with as time- varying covariates. The multivariable Cox regression analysis was performed using variables with *p* values of 0.1 or less in the univariable Cox regression analyses. Because the initial dosage and total dosage of paclitaxel contained overlapping information, each multivariable Cox regression model is presented separately (model 1, model 2) for the CLI subgroup analysis. To determine the optimal cut-off value of paclitaxel for predicting mortality, we followed the minimum *p* value approach. This was performed to select the optimal cut-off value that corresponds to the smallest *p* value derived from the univariate cox regression analysis between two groups by dividing above and below potential cut-off values. The cut-off value was analyzed between 5–40 mg, which is considered to be a meaningful range excluding the 10% range of upper and lower values from the investigated total cumulative paclitaxel dosage. The adjustment method by Benjamini and Hochberg (1995) was applied to prevent the increment of false positive error rate due to the multiple testing problem during the process of minimum *p* value approach. The level of statistical significance was set at *p* < 0.05. The analyses were performed with help from the statistics experts on the statistics support team at Samsung Medical Center.

## 3. Results

Among the 226 patients who underwent DCB angioplasty for femoropopliteal steno-occlusive disease between February 2013 and December 2018, 225 patients (mean age 71.2 ± 9.3 years, range 38–93 years, male 81%, 252 target limbs, 290 target lesions) were included in this analysis.

### 3.1. Demographics, Clinical Characteristics, and Paclitaxel Dosages

Table 1 summarizes the demographics, clinical characteristics, and paclitaxel dosages of all the patients. As noted, 149 patients (66.2%) were treated for claudication, and 76 patients (33.8%) were treated for CLI. Patients with CLI showed significantly higher mortality after DCB use than claudicants (66.07% vs. 23.08%, *p* < 0.001). In our study, 20.8% of the patients were diagnosed with cancer, among whom nine patients (18.2%) were newly diagnosed with cancer after their initial DCB angioplasty, and 20 patients (40.9%) were active cancer patients requiring treatment or follow up. The mean initial dosage of paclitaxel was 9.08 ± 5.72 mg (range 1.97–36.41 mg). This was higher in the patients who died, but the difference between those who died during follow up and those who survived was not statistically significant (10.37 mg vs. 8.65 mg; *p* = 0.066). Moreover, 23.7% of patients were treated with DCBs repeatedly during the follow-up period, and the mean frequency of DCB use was 1.28 ± 0.60 per patient. The frequency did not differ significantly between those who died during follow up and those who survived. The total dosage of paclitaxel was 11.70 ± 8.55 mg (range 1.97–52.37 mg) per patient, and it was higher in patients who died during follow up than in those who survived (14.05 mg vs. 10.92 mg, *p* = 0.023).

### 3.2. Causes of Death

From February 2013 to July 2020 (mean follow-up 35.1 ± 18.0 months, range 1–89 months), 56 patients (24.9%) died. As shown in Table 2, the most common cause of death was malignancy (*n* = 8, 14.3%), followed by infection, such as respiratory failure with pneumonia (*n* = 7, 12.5%) and septic shock (*n* = 7, 12.5%). Acute myocardial infarction or heart failure (*n* = 5, 8.9%) and sudden cardiac arrest (*n* = 4, 7.1%) caused 16% of all deaths during the study period. There was no 30-day mortality in this study. The mean period from the index treatment to death was 22.5 months (range 1–72.3 months).

### 3.3. Predictors of Mortality after DCB Treatment

Table 3 and Table 4 show the univariable and multivariable Cox regression models, respectively, for the predictors of mortality during follow up. The multivariable Cox regression analysis identified age (hazard ratio, HR, 1057; 95% confidence interval, CI, 1019–1096; *p* = 0.0032), CLI (HR, 11.487; 95% CI, 2.498–52.823; *p* = 0.0017), and the total dosage of paclitaxel (mg) (HR, 1040; 95% CI, 1006–1.074; *p* = 0.0210) as independent predictors of all-cause mortality during follow up. The initial dosage of paclitaxel (*p* = 0.8270) did not correlate with mortality during follow up. Although the frequency of DCB use was a significant factor in all-cause death in the univariable Cox analysis, the multivariable Cox regression model showed that the frequency of DCB use (*p* = 0.8981) did not predict mortality after correction for other factors. To evaluate potential interactions between the indication (claudication vs. CLI) and dosage of paclitaxel or frequency of DCB use, we analyzed the interaction effect. As shown in Supplementary Table S1, no interactions were significant.

### 3.4. Subgroup Analyses for Predictors of Mortality in the Claudication and CLI Groups

In the subgroup analysis of the claudication group, chronic kidney disease requiring hemodialysis (HR, 4.421; 95% CI, 1250–15.637, *p =* 0.0211) and cancer (newly diagnosed or active state) (HR, 5690; 95% CI, 2013–16.087; *p* = 0.0010) were identified as predictors of mortality during follow up. In the claudication group, the frequency of DCB use and total dosage of paclitaxel had no correlation with mortality in the multivariable analysis (frequency of DCB use, *p* = 0.3168; total dosage of paclitaxel, *p* = 0.0722) (Supplementary Table S2). In the CLI group, hyperlipidemia, chronic kidney disease, previous peripheral revascularization history, TASC classification, and the total dosage of paclitaxel (mg) (HR, 1046; 95% CI 1.007–1087; *p =* 0.0198) were identified as predictors of mortality during follow up in the multivariable Cox regression model (Supplementary Table S3).

### 3.5. Analyses for Optimal Cut-off Value of Total Paclitaxel Dosage for Predicting Mortality

**Figure 1:**
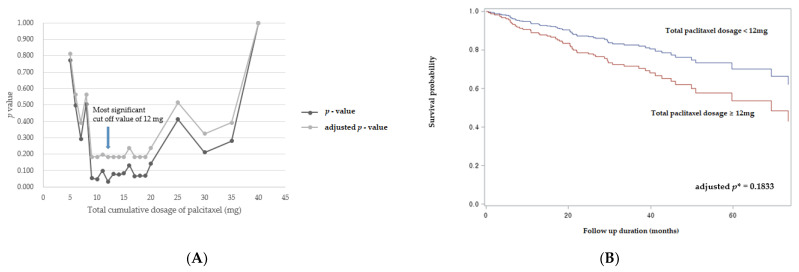
(**A**) A plot of *p* value and adjusted *p* value against candidate cut-off value of total cumulative paclitaxel dosage: Arrow indicates the most significant cut-off value as 12 mg; (**B**) Survival curve for the patient group based on the above and below cut-off value of total cumulative paclitaxel dosage, * by Benjamini & Hochberg method.

To determine the optimal cut-off value of paclitaxel for predicting mortality, we followed the minimum *p* value approach. Figure 1 presents a plot of the *p* value and adjusted *p* value against the candidate cut-off value of total cumulative paclitaxel dosage. The most significant cut-off value of total cumulative paclitaxel dosage for predicting mortality was 12 mg (*p*= 0.0339, adjusted *p =* 0.1833 by Benjamini–Hochberg method).

## 4. Discussion

In their systematic review and meta-analysis of RCTs, Katsanos and colleagues [2] raised a safety concern about two- and five-year mortality after the use of paclitaxel-coated devices. Moreover, they found that exposure to paclitaxel (dosage–time product) caused a 0.4% excess risk of death per paclitaxel mg-year [2]. According to Hill’s criteria for causation [9], if an agent causes an event, there should be a dose–response relationship. However, the Katsanos study was criticized for using published summary-level data, i.e., they estimated paclitaxel exposure rather than actually calculating individual patient dosages. A meta-analysis of individual patient data subsequently published by Vascular Interventional Advances(VIVA) physicians found a higher mortality rate with paclitaxel-coated devices but found no dose–mortality association [10]. Meanwhile, a recently published interim analysis of all-cause mortality in the Swedish Drug-elution Trial in Peripheral Arterial Disease (SWEDEPAD) registry RCT reported no significant difference in all-cause mortality between patients treated with paclitaxel-coated devices and those treated with uncoated devices [4]. However, the SWEDEPAD study used predominantly low dose paclitaxel–coated balloons and did not consider the cumulative paclitaxel doses from repeated use. Considering inferior clinical outcomes in the real-word data compared with RCTs [11] and the requirement for repeated use of DCBs for secondary revascularization procedures, a detailed review of patient-level data in real word experience is required to verify the mortality signal especially dose–response relationship. In this study, we investigated the correlation between the level of paclitaxel exposure and mortality using independent patient-level data from real-world experience.

Our study examined 225 patients who underwent DCB angioplasty for femoropopliteal steno-occlusive disease. We analyzed predictors of mortality using Cox regression models that considered comorbidities, paclitaxel dosages (initial and cumulative), and frequency of DCB treatment. The multivariable Cox regression analysis identified age, CLI, and total dosage of paclitaxel (mg) as predictors of mortality during follow up. Although the frequency of DCB use was statistically significant in the univariate analysis, it was not a significant predictor of mortality in the multivariate analysis after correction for other variables. However, the total dosage of paclitaxel was a predictor of mortality in the all-patient group and the CLI subgroup. Thus, the total cumulative dosage of paclitaxel correlates with the survival status of DCB angioplasty patients.

After the results of the Katsanos study were published, several patient-level meta-analyses and nationwide cohort studies were published. A meta-analysis of the IN.PACT Admiral DCB studies by Schneider et al. [5] reported no significant difference (*p =* 0.092) in the five-year mortality of the DCB (15.1%) and control groups (11.2%) in their patient population, and they found no dose–response relationship between paclitaxel and increased mortality. In addition, several real-world studies using mostly administrative databases [6,7] also reported finding no significant mortality difference between the DCB and uncoated PTA groups during follow-up, nor did they find a correlation between the paclitaxel dosage and mortality.

However, Rocha-Singh et al. [10] conducted a patient-level meta-analysis of 2185 patients from eight RCTs and found a 38% increase in relative five-year mortality risk, corresponding to a 4.6% absolute increase, associated with DCB use. Furthermore, Katsanos published another meta-analysis data investigating the treatment of infra-popliteal arteries using paclitaxel-coated balloons for CLI [12]. They again reported an increased risk of death and major limb amputation during one year of follow up in patients treated with a paclitaxel-coated device, with a higher risk found in patients treated with high-dose devices (3.0–3.5 μg/mm^2^) than in those treated with low-dose devices (2.0 μg/mm^2^). Subsequently, they published an updated systemic review and meta-analysis about the risk of major amputation after use of DCB in the femoropopliteal and infrapopliteal arteries [13]. In that report, the application of DCB was associated with a significantly higher risk of major amputation and this finding mostly affected the CLI patients, similar to the results of this study. They also reported that there was a significant dose-dependent association between cumulative paclitaxel dose and risk of major amputation [13].

In most published observational studies and RCTs that raise important mortality concerns about paclitaxel, patients with claudication accounted for more than 90% of the study populations, and the number of deaths was small. Therefore, the statistical power of the previously published results has been insufficient to show the real effect of paclitaxel on mortality. In our study, on the other hand, the high-risk CLI patient group accounted for about 34% of the total study population, which accurately reflects real-world practice. Moreover, we were able to completely investigate almost all of the deaths in our study population using data from the National Health Insurance Service and a telephone survey (97.8% survey response rate), and the death rate in our population was high, about 25%. Our findings, with our very low follow-up losses and high number of events, are thus significant because they reflect reliable data about the actual death rate.

Although several RCTs showed higher patency rates after DCB angioplasty than after conventional PTA [14,15,16], real-world clinical outcomes have been inferior to those reported in RCTs [11]. We found patients who required target lesion revascularization after DCB angioplasty or required angioplasty at another lower-extremity site and therefore underwent repeated angioplasty using DCBs during follow up. Therefore, the repeated use of DCBs and the cumulative dosage of paclitaxel must be taken into account when identifying correlations between dosage and mortality. Moreover, because the nominal paclitaxel dosage per balloon has a wide range depending on the length and diameter of the balloon (e.g., IN.PACT Admiral 1.089–6.897 mg; Lutonix 1.0–9.7 mg), and the number of DCBs used varies depending on the severity of the lesion, it is important to calculate the dosage of paclitaxel accurately for each patient. In this study, we found a wide range of initial (1.97–36.41 mg) and total (1.97–52.37 mg) dosages of paclitaxel per patient. Additionally, 23.7% of our patients were repeatedly treated with DCBs, with 5.8% of our patients being treated with a DCB more than three times during the follow-up period.

Although the patient-level meta-analyses presented so far have reflected the nominal dosage per patient, no previous studies have analyzed the cumulative total dosage of paclitaxel during follow up. We investigated the frequency of DCB use and total dosage of all DCBs by reviewing the procedural details for all patients, and we considered them as time-varying covariates to improve the accuracy of our analyses. After an additional analysis of the interaction effect between the indication and the dosage or frequency of DCB use, we confirmed that the total dosage of paclitaxel was an independent predictor of all-cause mortality during follow up. Although adjusted *p* value was not statistically significant due to the small sample size and effect size, we could suggest a meaningful cut-off value of paclitaxel dosage of 12 mg for predicting mortality. This result provides more convincing evidence of a dose–response relationship between paclitaxel and mortality than the results of previous research. We cannot provide a definitive cause of death or physiologic mechanism for the long-term fatal effects of paclitaxel in this study but systemic release and non-target embolization of cytotoxic paclitaxel particles in combination with the underlying ischemia and inflammation can be considered as a possible mechanism for potential adverse event as suggested by Katsanos [12,13]. A further study using a larger sample and prospectively collected data is needed to provide the definite causal mechanism for mortality. Nonetheless, we recommend the cautious use of DCB devices, and it might be necessary to consider another treatment option such as bypass surgery rather than use DCB angioplasty repeatedly, especially in CLI patients.

### Study Limitations and Significance

This study is limited by its retrospective, single-center design, relatively small sample size, significant differences in baseline characteristics, and inability to assess changes in medical treatment during the study period. It does not compare mortality rates between patients treated with uncoated PTA and those treated with DCBs. The individual causes of death could not be investigated in some patients, and we cannot definitively show the physiologic mechanism by which paclitaxel leads to death. Therefore, we cannot prove that the annual mortality risk in patients treated with paclitaxel DCB is increased. Although we found the cut-off value of paclitaxel for predicting mortality, we could not obtain a statistical difference between the two groups based on this cut-off value because of sample size and effect size. Nevertheless, we did examine real-world, patient-level data from a population that included about 30% CLI patients, and we completely investigated almost all deaths in the study population, which had a death rate of 25%. Furthermore, we were able to use precise procedural details, such as the frequency of DCB use and the cumulative total dosage of paclitaxel during follow up, in our regression analyses. The significance of our analysis is that it provides reliable results about the dose–response relationship, which are important in identifying the mortality signal of paclitaxel.

## 5. Conclusions

In real-world, patient-level data, the total cumulative dosage of paclitaxel from the use of paclitaxel-coated balloons correlated with all-cause mortality during follow up, especially in CLI patients. Our results provide limited information about the dose–response relationship between paclitaxel and mortality. This study does not provide direct evidence of a mortality difference between patients treated with uncoated PTA and those treated with DCBs, so a prospective, comparative study of long-term mortality after treatment with DCBs is required.

We investigated the correlation between the dosage and frequency of paclitaxel exposure and mortality using real-world, patient-level data from a population that included a large proportion of critical limb ischemia patients. Furthermore, we were able to use precise procedural details, such as the frequency of DCB use and the cumulative total dosage of paclitaxel during follow up. Our analysis provides reliable results about the dose–response relationship in paclitaxel-associated mortality concerns.

## Figures and Tables

**Table 1:** Baseline characteristics of all patients who survived versus those who died after treatment with paclitaxel-coated balloons.

Patient Characteristics	Survived (*n* = 169)	Died (*n* = 56)	*p*
Age, years (mean ± SD, range)	69.57 ± 8.90 (38–88)	76.27 ± 8.84 (55–93)	<0.001 *
Male (%)	140 (82.84)	42 (75.00)	0.196 **
Hypertension	139 (82.25)	48 (85.71)	0.549 **
Diabetes	117 (69.23)	43 (76.79)	0.280 **
Coronary artery disease	74 (43.79)	22 (39.29)	0.555 **
Smoking	76 (44.97)	16 (28.57)	0.031 **
Smoking (current)	48 (28.40)	12 (21.43)	0.306 **
Chronic kidney disease	62 (36.69)	38 (67.89)	<0.001 **
On hemodialysis	19 (11.24)	15 (26.79)	0.005 **
Hyperlipidemia	87 (51.48)	17 (30.36)	0.006 **
Cerebrovascular attack	35 (20.71)	10 (17.86)	0.644 **
Chronic obstructive pulmonary disease	2 (3.57)	9 (5.33)	0.736 †
Cancer	30 (17.75)	17 (30.36)	0.044 **
Cancer newly diagnosed after treatment	5 (2.96)	4 (7.14)	
Active cancer	13 (7.69)	7 (12.50)	
Cured of cancer	12 (7.10)	6 (10.71)	
Previous peripheral revascularization	20 (11.83)	12 (21.43)	0.160 †
Treatment indication			
Claudication	130 (76.92)	19 (33.93)	<0.001 **
Critical limb ischemia	39 (23.08)	37 (66.07)	
TASC classification			
A	44 (26.04)	13 (23.21)	0.967 †
B	93 (55.03)	32 (57.14)	
C	24 (14.20)	8 (14.29)	
D	8 (4.73)	3 (5.36)	
F/U duration, months			
Mean ± SD	39.81 ± 16.60	22.77 ± 17.14	<0.001 *
Initial dosage of paclitaxel (mg)			
Mean ± SD	8.65 ± 5.29	10.37 ± 6.75	0.066 *
Median (Q1, Q3)	6.95 (4.75, 10.43)	8.45 (5.12, 13.60)	
Min, Max	1.97, 28.16	3.00, 36.41	
Total dosage of paclitaxel (mg)			0.023 *
Mean ± SD	10.92 ± 7.94	14.05 ± 9.88	
Median (Q1, Q3)	8.60 (5.81, 13.61)	10.43 (6.92, 19.15)	
Min, Max	1.97, 52.37	3.00, 43.46	
Repeated use of DCB			
Yes	37 (21.89)	16 (28.57)	0.307 **
No	132 (78.11)	40 (71.43)	
Frequency of DCB use			0.162 *
Mean ± SD	1.27 ± 0.59	1.34 ± 0.61	
1	135 (79.88)	41 (73.21)	
2	25 (14.79)	11 (19.64)	
3	7 (4.14)	4 (7.14)	
4	2 (1.18)	0	

SD, standard deviation; F/U, follow up; DCB, drug coated balloon; TASC, Trans Atlantic Inter-Society Consensus; * Mann-Whitney test; ** chi-square test; † Fisher’s exact test.

**Table 2:** Causes of death (*N* = 56 patients).

Cause of Death	*N* (%)
Malignancy	8 (14.3)
Lung cancer	2 (25.0)
Lymphoma	2 (25.0)
Bladder cancer	1 (12.5)
Pancreas cancer	1 (12.5)
Colon cancer	1 (12.5)
Glottic cancer	1 (12.5)
Respiratory failure with pneumonia	7 (12.5)
Septic shock	7 (12.5)
Acute MI or heart failure	5 (8.9)
Sudden cardiac arrest	4 (7.1)
Senility	2 (3.6)
Gastrointestinal bleeding	1 (1.8)
Cerebral infarction	1 (1.8)
Brain hemorrhage	1 (1.8)
Undetermined	20 (35.7)

MI, myocardial infarction.

**Table 3:** Univariable Cox regression analyses of predictors for all-cause death.

Effect		Hazard Ratio	95% CI		*p*-Value	Type 3 Analysis of Effects *p*-Value
Sex (Female)		1.525	0.832	2795	0.1721	0.1721
Age		1.086	1049	1124	<0.0001	<0.0001
Critical limb ischemia		6.040	3362	10.854	<0.0001	<0.0001
Diabetes		1.581	0.847	2.951	0.1505	0.1505
Hypertension		1.303	0.616	2.756	0.4889	0.4889
Hyperlipidemia		0.446	0.252	0.790	0.0056	0.0056
Coronary artery disease		0.823	0.481	1.407	0.4756	0.4756
Smoking		0.527	0.294	0.943	0.0310	0.4756
Smoking (current)		0.748	0.393	1.422	0.3754	0.3754
Chronic kidney disease		2.881	1643	5.052	0.0002	0.0002
Chronic kidney disease on HD		2.723	1498	4.949	0.0010	0.0010
Cerebrovascular attack		0.831	0.419	1.647	0.5957	0.5957
Chronic obstructive pulmonary disease		0.766	0.186	3.146	0.7112	0.7112
Cancer *		2.159	1111	4.197	0.0233	0.0233
Previous peripheral revascularization		1.638	0.863	3.109	0.1313	0.1313
TASC	B vs. A	1.036	0.471	2.280	1.0000	0.9529
	C vs. A	0.837	0.283	2.472	1.0000	
	D vs. A	1.131	0.243	5.258	1.0000	
Initial dosage of paclitaxel (mg)		1.032	0.992	1.073	0.1238	0.1238
Total dosage of paclitaxel (mg) **		1.040	0.996	1.086	0.0731	0.0731
Frequency of DCB use^**^		2.021	1351	3.022	0.0006	0.0006

* Newly diagnosed after initial treatment or active state that required treatment or follow up; ** Time-varying covariate; HD, hemodialysis; DCB, drug coated balloon. CI: confidence interval

**Table 4:** Multivariable Cox regression analysis of predictors for all-cause death.

Effect	Adjusted Hazard Ratio	95% CI		*p*-Value
Age	1.057	1.019	1.096	0.0032
Critical limb ischemia	4.135	2.171	7.876	<0.0001
Hyperlipidemia	0.545	0.295	1.007	0.0526
Smoking	0.993	0.523	1.886	0.9825
Chronic kidney disease	1.784	0.933	3.410	0.0799
Chronic kidney disease on HD	1.340	0.635	2.830	0.4429
Cancer *	1.189	0.583	2.427	0.6336
Frequency of DCB use **	1.031	0.647	1.643	0.8981
Total dosage of paclitaxel (mg) **	1.040	1.006	1.074	0.0210

* Newly diagnosed after initial treatment or active state that required treatment or follow up; ** Time-varying covariate; HD, hemodialysis; DCB, drug coated balloon.

## Supplementary Materials

The following are available online at www.mdpi.com/article/10.3390/jcm10163747/s1, Table S1: The interaction effects among variables used in the multivariable analysis, Table S2: Univariable (**A**) and multivariable (**B**) Cox regression analysis of the predictors of all-cause death in the claudication group, Table S3: Univariable (**A**) and multivariable (**B**) Cox regression analysis of the predictors of all-cause death in the critical limb ischemia group.
