# Supplementary material for: Mortality after Use of Paclitaxel-Coated Balloons Correlates with Total Cumulative Dosage of Paclitaxel in Real-World Analysis
Source: J Clin Med. 2021 Aug 23;10(16):3747. doi: 10.3390/jcm10163747 (PMC8396966; doi:10.3390/jcm10163747)
Supplement: Supplementary file 1 [file jcm-10-03747-s001.zip › jcm-1347381-SI.pdf]

Table S1. The interaction effects among variables used in the multivariable analysis.

**Effect.**	***p*-value**
Age	0.0046
Hyperlipidemia	0.0679
Smoking	0.9143
Chronic kidney disease	0.2198
Chronic kidney disease on hemodialysis	0.2936
Cancer	0.8822
Critical limb ischemia (CLI)	0.0011
Frequency of DCB use	0.2181
CLI * Frequency of DCB use	0.1832
Total dosage of paclitaxel	0.0928
CLI * Total dosage of paclitaxel	0.9437

CLI, critical limb ischemia; DCB, drug-coated balloon.

Table S2. Univariable (**A**) and multivariable (**B**) Cox regression analysis of the predictors of all-cause death in the claudication group.

(A)

**Effect**		**Hazard Ratio**	**95% CI**		***p*-value**	**Type 3 Analysis of Effects *p*-value**
Gender_Female		0.364	0.049	2.728	0.3254	0.3254
Age		1.055	0.994	1.120	0.0789	**0.0789**
Diabetes		1.329	0.501	3.524	0.5680	0.5680
Hypertension		0.695	0.250	1.938	0.4873	0.4873
Hyperlipidemia		0.810	0.329	1.999	0.6480	0.6480
Coronary artery disease		1.457	0.591	3.591	0.4136	0.4136
Smoking		1.092	0.439	2.721	0.8495	0.8495
Smoking_current		1.293	0.475	3.522	0.6151	0.6151
Chronic kidney disease		1.783	0.720	4.417	0.2115	0.2115
Chronic kidney disease on HD		4.010	1.305	12.323	0.0153	**0.0153**
Cerebrovascular attack		0.499	0.115	2.162	0.3528	0.3528
Chronic obstructive pulmonary disease		0.436	0.024	7.888	0.5743	0.5743
Cancer*		5.585	2.054	15.191	0.0008	**0.0008**
Previous peripheral revascularization		1.257	0.413	3.827	0.6866	0.6866
	B vs.. A	5.514	0.459	66.278	0.3006	
TASC	C vs.. A	3.951	0.270	57.914	0.6618	0.4047
	D vs.. A	3.505	0.118	104.231	1.0000	
Initial dosage of paclitaxel (mg)		1.044	0.982	1.110	0.1693	0.1693
Total dosage of paclitaxel (mg) **		1.067	1.028	1.107	0.0006	**0.0006**
Frequency of DCB use**		3.864	2.005	7.447	<0.0001	**<0.0001**

*: Newly diagnosed after initial treatment or active state that required treatment or follow up; **: time-varyingcovariate. HD, hemodialysis; DCB, drug-coated balloon; CI: confidence interval; TASC, Trans Atlantic Inter-Society Consensus

(B)

Effect	Adjusted Hazard Ratio	95% CI		*p*-value
Age	1.045	0.982	1.113	0.1676
Chronic kidney disease on HD	4.465	1.282	15.554	0.0188
Cancer*	4.834	1.671	13.982	0.0036
Frequency of DCB use **	1.427	0.711	2.864	0.3168
Total dosage of paclitaxel (mg) **	1.043	0.996	1.092	0.0722

* Newly diagnosed after initial treatment or active state that required treatment or follow up; ** time-varying covariate. HD, hemodialysis; DCB, drug-coated balloon.

Table S3. Univariable (**A**) and multivariable (**B**) Cox regression analysis of the predictors of all-cause death in the critical limb ischemia group.

(A)

Effect		Hazard Ratio	95% CI		*p*-value	Type 3 Analysis of Effects *p*-value
Gender_Female		1.168	0.594	2.296	0.6519	0.6519
Age		1.067	1.027	1.108	0.0009	0.0009
Diabetes		1.210	0.529	2.768	0.6523	0.6523
Hypertension		1.415	0.434	4.617	0.5649	0.5649
Hyperlipidemia		0.394	0.180	0.864	0.0200	0.0200
Coronary artery disease		0.631	0.316	1.259	0.1914	0.1914
Smoking		0.477	0.209	1.092	0.0799	0.0799
Smoking_current		0.735	0.306	1.764	0.4907	0.4907
Chronic kidney disease		2.322	1.092	4.938	0.0287	0.0287
Chronic kidney disease on HD		1.229	0.606	2.493	0.5668	0.5668
Cerebrovascular attack		0.833	0.380	1.826	0.6478	0.6478
Chronic obstructive pulmonary disease		2.161	0.517	9.036	0.2912	0.2912
Cancer*		0.974	0.379	2.504	0.9569	0.9569
Previous peripheral revascularization		4.226	1.901	9.397	0.0004	0.0004
	B vs. A	0.587	0.242	1.423	0.4497	
TASC	C vs. A	1.032	0.257	4.143	1.0000	0.0284
	D vs. A	5.414	0.799	36.687	0.1038	
Initial dosage of paclitaxel (mg)		1.063	1.002	1.128	0.0437	0.0437
Total dosage of paclitaxel (mg) **		1.036	0.997	1.078	0.0743	0.0743
Frequency of DCB use**		1.042	0.591	1.838	0.8872	0.8872

* Newly diagnosed after initial treatment or active state that required treatment or follow up; ** time-varying covariate; HD, hemodialysis; DCB, drug-coated balloon.

(B)

(Model 1)

**Effect**		**Adjusted Hazard Ratio**	**95% CI**		***p*-value**	**Type 3 Analysis of Effects *p*-value**
Age		1.037	0.995	1.082	0.0874	0.0874
Hyperlipidemia		0.495	0.209	1.173	0.1101	0.1101
Smoking		0.705	0.285	1.748	0.4511	0.4511
Chronic kidney disease		3.299	1.366	7.967	0.0080	0.0080
Previous peripheral revascularization		4.068	1.718	9.635	0.0014	0.0014
	B vs. A	0.421	0.153	1.158	0.1221	
TASC	C vs. A	0.565	0.131	2.440	1.0000	0.0109
	D vs. A	4.980	0.513	48.376	0.2730	
Initial dosage of paclitaxel (mg)		1.053	0.987	1.124	0.1170	0.1170

(Model 2)

**Effect**		**Adjusted Hazard Ratio**	**95% CI**		***p*-value**	**Type 3 Analysis of Effects *p*-value**
Age		1.039	0.995	1.084	0.0800	0.0800
Hyperlipidemia		0.400	0.166	0.964	0.0412	0.0412
Smoking		0.698	0.281	1.734	0.4389	0.4389
Chronic kidney disease		3.815	1.565	9.296	0.0032	0.0032
Previous peripheral revascularization		4.159	1.751	9.881	0.0012	0.0012
	B vs. A	0.461	0.177	1.199	0.1572	
TASC	C vs. A	0.521	0.120	2.252	0.8583	0.0100
	D vs. A	5.818	0.622	54.433	0.1782	
Total dosage of paclitaxel (mg)*		1.046	1.007	1.087	0.0198	0.0198

* Time-varying covariate.
